# 4Ps framework: practical actions to protect individual health in the current climate crisis

**DOI:** 10.1136/leader-2025-001262

**Published:** 2025-09-04

**Authors:** Hugh Montgomery, Amir Baniassadi, Wenjia Cai, Ali Kubba, Li Li, Rossella Nappi, Amanda Stucke

**Affiliations:** 1 Department of Medicine, University College London, London, England, UK; 2 Department of Medicine, Harvard Medical School, Boston, Massachusetts, USA; 3 Hinda and Arthur Marcus Institute for Aging Research, Boston, Massachusetts, USA; 4 Department of Earth System Science, Tsinghua University, Beijing, Beijing, China; 5 Guy’s and St Thomas’ Hospitals NHS Trust, London, England, UK; 6 Bayer AG, Leverkusen, NRW, Germany; 7 Department of Clinical, Surgical, Diagnostic and Pediatric Sciences, University of Pavia, Pavia, Lombardia, Italy; 8 Research Center for Reproductive Medicine, Gynecological Endocrinology and Menopause, IRCCS Policlinico San Matteo, Pavia, Italy; 9 Economist Impact, London, England, UK

**Keywords:** Education, GP, healthcare planning, health policy, multi-disciplinary

## Abstract

Climate change driven by anthropogenic greenhouse gas (GHG) emissions represents an immediate and grave threat to human health and survival. Sea level rise, altered weather patterns and increasingly frequent and severe extreme weather events can damage health directly (eg, injury, heat stress, altered aeroallergen and particulate exposure). They also bring indirect health impacts through altered patterns of zoonotic and vectorborne diseases, disruption of food systems and downstream social consequences (economic collapse, mass migration and conflict).

Healthcare providers and healthcare workers all need to take immediate action to drive and deliver reductions in GHG emissions, and to help patients in better managing the health impacts brought about by climate change. Here, we propose the ‘4Ps framework’ (Personal, Professional, Pathway-specific and Policy) to empower and facilitate such action.

## Introduction

 The stability of the Earth’s climate over the past 11 700 years has allowed human civilisation to flourish.^1^ Greenhouse gases (GHGs), such as carbon dioxide (CO_2_) and methane, allow shortwave (solar) radiation to pass through them but trap longwave radiation in the form of heat. Combustion of fossil fuels (as well as deforestation, changes in agriculture and emission of some synthesised GHGs) is increasing atmospheric GHG concentrations, meaning that our planet is gaining more energy than it loses to space. Climate change threatens our health and survival—WHO declared it to be the single biggest health threat facing humanity.^2^ Indeed, the global ecosystem upon which our species’ survival depends is under immediate threat.^3–5^ According to the Intergovernmental Panel on Climate Change, “any further delay in concerted global action will miss a brief and rapidly closing window to secure a liveable future”.^6^

Healthcare providers (HCPs) and healthcare workers (HCWs) around the world are well placed to

- Understand the impact which climate change poses to human health and survival.
- Drive meaningful action to reduce GHG emissions.
- Assist patients in adapting to the changes that are already happening.

### Conventional health considerations

Health is affected by climate change in myriad ways. Elevated temperatures (both sustained and/or sudden increases), air pollution (aeroallergens, pollutant gases and particulates from fossil fuel combustion and wildfires), altered patterns of zoonotic and vectorborne diseases, and disruption to food systems all impact individuals’ physical and mental health.^7^ Heat stress increases the risk of cardiovascular events and kidney disease.^8^ Gaseous and particulate pollutants and changes in allergen and dust exposure cause exacerbations of asthma and chronic obstructive pulmonary disease (COPD).^9^ The burden of vectorborne diseases increases due to more geographical areas supporting vector survival, increases in feeding and breeding patterns, and higher pathogen replication rates.^10^ Changes in the ecosystem force animals and humans into greater proximity, increasing the risk of animal-to-human transmission of infectious agents.^11^ Weather-induced damage to sewage infrastructure also leads to microbial contamination of wastewater and thence of soil and drinking water, all resulting in associated diseases. Sea level rise also leads to saltwater contamination of soil and drinking water.

The conventionally considered impacts of climate change and health are disproportionately felt across populations: variation in the social determinants of health results in differences in exposure, sensitivity and adaptive capacity.^12^ The greatest impacts are thus often first felt in ethnic minorities, Indigenous peoples, low-income communities, migrants and displaced people, as well as women experiencing pregnancy and childbirth.^12^ But none are immune, regardless of wealth or location.

### Socio-economically mediated impacts of climate change on health and survival

While these pathophysiological impacts of climate change have been a major focus, the health and survival impacts mediated by socio-economic consequences have been far less considered.

Global economic losses of US$5 trillion are plausible in the next 4 years, with a 1:300 chance of the global economy losing US$17.6 trillion in a single extreme weather event.^13^ Regardless of future emission choices, the world economy will suffer an income loss of US$38 trillion/year (and perhaps as much as US$59 trillion/year) within 26 years.^14^ Current climate policies risk “catastrophic societal and economic impacts”,^15^ and the Institute and Faculty of Actuaries has warned that our economy “may not exist at all” if climate change is not mitigated through transformative (and not incremental) action.^16^

Such economic collapse drives mass migration/displacement and conflict/war, as do many of the other impacts of climate change (such as disease, sea level rise, lack of food and loss of habitation).^17^ Global heating is now accelerating fast, and, without immediate action, irreversible collapse (eg, in regional, and then global, biospheres and major ocean circulations) will be seen by current generations.^18^

There is a growing body of literature and guidance on actions that HCPs and HCWs can take to mitigate GHG emissions and therefore protect themselves and their patients. However, most publications relate solely to ‘reducing health service emissions’ (often in particular parts of services) and do not cover every element of action with which any one individual can engage. A framework applicable to all different areas of putative action might thus empower HCPs and HCWs further.

### A framework for action

**Figure 1:**
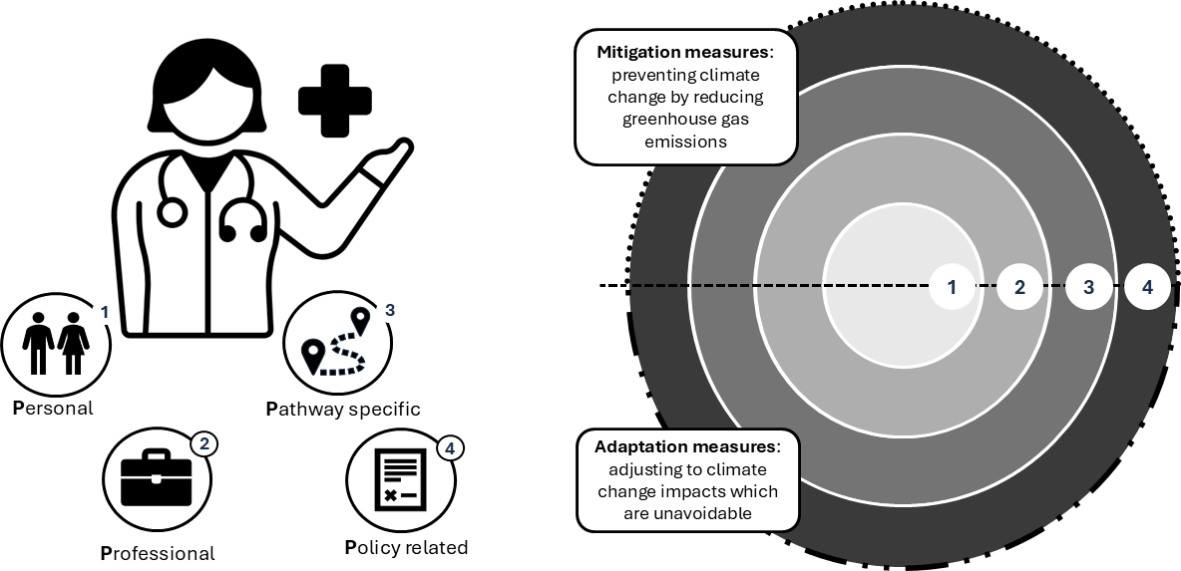
The Personal, Professional, Pathway-specific and Policy (4Ps) framework for practical actions healthcare practitioners and healthcare workers can take to protect individuals and society from worsening climate change.

GHGs can have a very long atmospheric lifespan, meaning that the impact of emissions today can reach far into the future—one-fifth of the CO_2_ we emit today will still be heating the planet in 33 000 years’ time.^19^ Actions at pace and scale to reduce emissions are thus necessary so as to reduce their temporal ‘area under the curve’ dramatically, and many such actions also bring direct health co-benefits.^20^ While various initiatives for HCW and HCP engagement have been proposed,^21^ there has been minimal guidance on the practical actions that they can take. Here, we propose ‘*The 4Ps Framework’* of Personal, Professional (advice to patients and colleagues, changes in workplace or business operation), Pathway-specific (activities related to disease management) and Policy (relate to local health regulators, partnerships or care providers; professional bodies; healthcare charities; and local, national and international government structures) actions to assist them in making a meaningful difference (figure 1).

## The 4Ps framework: Personal, Professional, Pathway-specific and Policy

### Mitigation and adaptation

Actions to reduce the health impacts of climate change may relate to mitigation (preventing climate change by reducing GHG emissions) or adaptation (adjusting to unavoidable climate change impacts). Since the impacts of climate change may soon exceed the capacity for adaptation, it is vital that mitigative actions are initiated. Furthermore, reducing GHGs can save healthcare costs—for every dollar spent, approximately $2 are saved in health costs.^22^

There are simple actions which HCPs and HCWs could perform to reduce their GHG emissions.^17^ At the same time, they can also help their patients manage the health impacts of climate change that are already being felt (table 1). While many of the actions are role-dependent, HCPs and HCWs are encouraged to build partnerships to facilitate wider impact.

**Table 1:** Mitigation and adaptation strategies for HCPs and HCWs to address climate change and its impacts on health

Personal	Professional advice to patients and colleagues	Pathway-specific activities related to disease management	Policy
Mitigation strategies to reduce greenhouse emissions and drawdown			
Consume plant-based diets, which are ideally also local and seasonal Travel better (active transport>mass public transport>shared private and limiting flying) Move money and investments from institutions that fund fossil-fuel extraction Buy less (eg, reduce heating/insulate better; purchase fewer products) Switch power supply to 100% renewable energy and electrify heating (heat pumps) Measure individual carbon footprint and address each element Generate less waste, reuse and recycle	Motivate, empower and support patients and colleagues to perform the ‘personal’ acts Inform and educate patients and colleagues about the effects of climate change on health Encourage hospitals to offer (and encourage staff and patients to consume) plant-based diets Find ways to reduce travel (and make it less carbon intensive) for patients and staff Review procurement policies and change contracts such that procurement moves to those companies that will decarbonise rapidly Switch to 100% renewable power supplies and reduce energy consumption in buildings by paying attention to energy efficiency/saving measures	Mirror the activities in the ‘personal’ acts in daily practice Focus on disease prevention and public health promotion Diagnose early, optimise management, and consider greener alternatives where available Avoid over-investigation; avoid unnecessary referrals, tests, invasive procedures, over-treatment, and medicine wastage	Advocate and support policies that encourage the actions mentioned in the ‘personal’ acts Inform and educate to engender and encourage action Encourage healthcare systems to measure and monitor their GHG emissions and act to rapidly reduce them Engage other health stakeholders (including allied health professionals), representative societies and bodies, healthcare funders, healthcare charities to take action and to engage their own members Engage local and national politicians and cultural and religious leaders
Adaptation strategies to help patients cope with the health impacts of climate change that are already being felt			
Educate themselves on the science and health impacts of climate change	Remind patients to speak regularly to their pharmacist and/or physician about their medications, especially before travel or when extreme weather events such as heatwaves are anticipated Advise patients on strategies to reduce their risks from heat and air pollution Educate pharmacists and patients on how to store climate-sensitive medications Use data to identify and intervene with at-risk populations Educate family members to check in with at-risk people regularly and during extreme weather events		Develop guidance on how to Protect individual health in extreme weather Adjust medication (or its intake/water intake) for specific diseases in extreme weather Develop and promote health education materials and policies that empower patients by increasing their awareness of climate-sensitive health conditions Improve and provide continued education for HCWs and HCPs on the impact of climate change

GHG, greenhouse gas; HCP, healthcare provider; HCW, healthcare workers.

## Mitigation measures

### Personal

- Consume plant-based diets (meat in the form of poultry or pork, whichever is most culturally acceptable, in smaller portions and less frequently, if at all), which are ideally also local and seasonal.
  Food production accounts for 34% of global GHGs and 73% of tropical and subtropical deforestation.^23^ Over 55% of anticipated heating can be avoided through simultaneous improvements in production practices, universal adoption of a healthy plant-based diet (such as the EAT-*Lancet* Commission’s Planetary Diet^24^) and consumer-level and retail-level food waste reductions.^25^ There are also direct health co-benefits to such a dietary change—reduction in the intake of saturated fat and ultraprocessed food can reduce the risk of cardiorenal metabolic conditions, hypertension, dementia, asthma and many cancers.^26^
- Engage in green travel.
  In 2022, the transport sector accounted for 8 billion tonnes of CO_2_ emissions and 14% of all GHG emissions worldwide.^27^ To reduce transport-related emissions, walk or cycle where possible; where this is not possible, mass public transport is preferred; and if necessary, car sharing is better than individual car journeys. Flying should also be minimised. Health co-benefits of active transport include reduced risks of cardiovascular disease, obesity, multiple cancers, osteoporosis, sarcopenia and dementia. In addition, emissions of toxic gaseous and particulate pollution can be reduced, which aids in lowering the prevalence of asthma, exacerbations of COPD, cancer (breast, bowel, etc), dementia and cardiovascular disease.

- Move money to a bank that does not fund fossil fuel extraction and likewise move any investments which fund such extraction. This removes the social licence for such funding.^28^
- Do less/buy less.
  Buy reused/recycled products where possible and choose the ‘greenest’ company or supplier.

- Switch power supply to 100% renewable energy.
  Where possible, purchase power from a ‘100% renewable’ supplier and/or from installed equipment for solar/wind power generation, as well as ground and air source heat pumps. By displacing fossil fuel combustion, renewable sources of energy reduce the rates of asthma and cardiovascular disease by mitigating air pollution from power generation. In addition, it is reported that 2.6 billion people in low-income and middle-income countries use highly polluting (and health-harming) solid fuel (eg, biomass and coal) stoves^29^; reduction in their use could bring both health and environmental benefits.
- Measure personal carbon footprint and act on each element.
  This can readily be done with a free online tool (eg, in the UK: https://www.carbonfootprint.com/calculator.aspx).
- Generate less waste, reuse and recycle.
  Different types of waste impact the environment to differing extents, but landfill and incineration both have a negative impact on emissions. Recycling alone could reduce emissions by 5.5 to 6.02 gigatonnes of CO_2_ between 2020 and 2050.^28^

### Professional actions

Motivate, empower and support patients and colleagues to perform the ‘personal’ acts. Some ways in which this can be achieved include

- Inform and educate patients and colleagues on the effects of climate change on health: Existing evidence supports the role of HCPs and HCWs in building support for climate action. Health professionals are highly trusted to talk about the health impacts of climate change and have considerable potential to build public support for climate solutions.^30^ Simple, jargon-free, protective rather than risk messages should be emphasised and often repeated when communicating the health impacts of climate change.^21 31^ In particular, vulnerable populations have been shown to perceive greater health risks from climate change^31^ ; these groups are often instrumental in bringing about change. The WHO toolkit provides useful guidance on how HCPs and HCWs can effectively communicate the health impacts of climate change.^21^

- Increase consumption of plant-based meals in hospitals: Plant-based diets (less meat in smaller quantities and avoiding red meat especially) are good for health and also reduce emissions from the food supply chain. For example, New York’s hospitals have moved to a ‘vegan-by-default’ menu with 92% patient approval, while also saving money (¢59 per meal) and carbon emissions (36%).^32^ Many other hospitals worldwide are also shifting to such action. Individuals can engage with their hospital management and catering services to encourage and support these initiatives. An article by Karlsen *et al* highlighted various strategies for practitioners to support patients in plant-based eating.^33^ HCPs involved in developing policies for facilities can support plant-based meals with scientific evidence.
- Find ways to reduce travel for patients and staff: A study conducted in the USA between 2022 and 2023 estimated that annual patient healthcare-related travel in the USA generated 35.7 megatonnes of CO_2_ equivalent emissions.^34^

Some ways to reduce travel include

- Offer online follow-ups, telemedicine and wearable devices.

- Reduce the frequency of follow-up visits.
- Combine testing and assessment appointments across disciplines to minimise visits.
- Reduce unnecessary tests at healthcare facilities.
- Establish protocols for self-testing or remote testing.
- Support staff active travel (walking and cycling), public transport and car-sharing.
- Transition to low-emission green ambulances and light vehicles.
- Discourage flights for speakers at meetings and other in-person fossil fuel-powered meeting attendance.

Since many of these suggestions are systemic, HCPs need to engage with senior management for decision-making and empower patients in behavioural change to promote these actions. There are many examples where such actions have been taken.^35–40^

- Wherever HCPs and HCWs are able to influence, or are directly involved in procurement, try to ensure that procurement comes from companies that will not decarbonise rapidly (ie, over 5 years or so).

Power purchase agreements with energy companies offer an additional way to save money and reduce emissions from power use (essentially ‘funding the build of a renewable energy facility’). For purchases, reusable instruments and protective clothing can be sought where possible and contracts written to prioritise purchases from ‘low carbon’ suppliers.

- Switch to clean energy sources and/or reduce energy consumption in buildings by paying attention to energy efficiency/saving measures. The healthcare sector is responsible for about 5.2% of global emissions. Implementing clean energy sources not only reduces GHG emissions but also improves the capacity of healthcare systems to manage disruptions from extreme weather events, pandemics and other health crises.^21^ HCPs and HCWs can take simple measures such as optimal use of lights, heating and cooling, and electronic equipment to reduce the carbon footprint in various facilities.

### Pathway-specific actions

Mirror the activities in the ‘personal’ acts in daily practice relating to disease management.

- Focus on disease prevention and health promotion.
  - Implement and promote appropriate health-improving actions (eg, smoking cessation, dietary change, exercise and active transport); ‘nature prescribing’ also links people to nature-based interventions and activities such as local walking as part of daily consultations.
- Diagnose early, optimise management and consider greener alternatives where available.
  - Where appropriate, use dry powder inhalers (DPIs) instead of pressurised metered-dose inhalers (MDIs): MDIs have>30 times larger carbon footprints than DPIs across their lifecycle.^41^
  - Follow, implement and enforce antimicrobial stewardship policies, depending on the role of HCPs and HCWs. There are different ways of doing this at different levels. In daily practice, HCPs are encouraged to switch from intravenous to oral antibiotics as soon as possible and use the shortest safe course. In one study across 610 general practices, a digital webinar followed by monthly reports and decision support tools to inform prescribing was shown to reduce antibiotic prescribing rates.^42^
- Avoid over-investigation, unnecessary referrals, tests, invasive procedures, over-treatment and medicine wastage.
  - Improve the efficiency of interventions. For example, one study found that implementing the ‘Choosing Wisely’ initiative, which promotes conversations between doctors and patients to avoid unnecessary interventions, reduced blood tests by 50% and the length of hospital stays by 20% in one inpatient setting.^43^ HCPs and HCWs can familiarise themselves with such initiatives and promote them in their setting depending on their roles.
  - Avoid polypharmacy, especially as certain medications may increase the health impacts of climate change.
  - Use medical imaging and laboratory testing judiciously.
  - Reduce use of unnecessary consumables (eg, non-sterile glove use) and use reusable items wherever possible.

### Policy-level actions

Advocate and support policies that encourage the actions mentioned in the above sections, including those listed under ‘personal’ actions.

- Inform and educate to engender and encourage action.
- Encourage healthcare systems to measure and monitor their GHG emissions through daily conversations or when contributing to policies. This allows organisations to develop and enact ways to reduce them. In the UK, for example, the environmental performance of National Health Service (NHS) organisations is monitored through the Greener NHS dashboard, which includes key indicators on anaesthetics, inhalers and building energy use. Furthermore, data from monitoring systems related to the impact on health are useful to the scientific and policymaking communities. Monitoring should include emissions related to Scope 1 (GHG release onsite, including volatile anaesthetic agents and those from fossil fuel combustion onsite), Scope 2 (emissions related to purchased power) and Scope 3 (related to procured materials, products, services and actions, including patient travel).

## Adaptation measures

### Personal

- HCPs and HCWs should educate themselves on the science and health impacts of climate change.
  Reputable sources include the Lancet Countdown on Health and Climate Change (https://www.thelancet.com/countdown-health-climate), the Centers for Disease Control and Prevention (www.cdc.gov) and the Environmental Protection Agency (www.epa.gov), although the latter may be removed by the current US administration.

### Professional and pathway-specific actions

- Remind patients to speak regularly to their pharmacist and/or physician about their medications, especially before travel or when extreme weather events such as heatwaves are anticipated.
  Some drugs/medications interfere with the body’s homeostatic responses to elevated temperatures, predisposing the individual to heat-related illnesses.^44^
- Advise patients on strategies to reduce their risks from heat and air pollution.
  For example, where possible:
  - During heatwaves, stay in cool indoor areas (which might include shopping malls) or under shade in the middle of the day and avoid physical labour and exercise. Maintain adequate water intake.
  - In areas of high pollution, walk away from main roads and use an N95 mask if particulate pollution is substantial; or use an air purifier indoors.
- Educate pharmacists and patients on how to store temperature-sensitive medications.
  Many medications lose their efficacy when stored at temperatures above 25°C.^45 46^ For example, the efficacy of PCSK9 inhibitors significantly decreases when they are stored at temperatures higher than 25°C for several hours.^47^
  Use data to identify and intervene with at-risk populations. Educate family members to check in with at-risk people regularly and during extreme weather events. While all individuals are at risk of poorer health outcomes from climate change, some are more susceptible to harm due to a combination of the limited resources available and the life challenges they face.^48^ The most vulnerable populations are those to which a negative determinant in more than one category applies.

### Policy-level actions

- HCPs and HCWs can contribute to developing
  - Guidance on protecting individual health in extreme weather (for vulnerable populations and the general public).
  - Guidance on adjusting medication (or its intake/water intake) for specific diseases in extreme weather.
  - Health education materials and policies that empower patients by increasing their awareness of climate-sensitive health conditions. For example, educational materials on different health conditions (posters/leaflets/podcasts, radio/television spots, etc) or offering micro-credentials to students or employers on specific topics.
- Improve and provide continued education for HCWs and HCPs on the impact of climate change.
  HCPs should advocate the widespread adoption of such curricula at medical schools. Some medical schools, such as the Harvard Medical School, are already integrating climate-related curricula for their medical students.^49^ The General Medical Council for the UK is working with the Planetary Health Report Card initiative to integrate this topic into the undergraduate curriculum.^50^

## Concluding remarks

Climate change poses an immediate threat to the survival of all those alive today. HCWs and HCPs can play a major role in mitigating this risk by using a simple ‘4Ps framework’ of practical action.

## Data Availability

No data are available.
